# Growth of the northeastern margin of the Tibetan Plateau by squeezing up of the crust at the boundaries

**DOI:** 10.1038/s41598-017-09640-0

**Published:** 2017-09-06

**Authors:** Jianyu Shi, Danian Shi, Yang Shen, Wenjin Zhao, Guangqi Xue, Heping Su, Yang Song

**Affiliations:** 1 MLR Key Laboratory of Metallogeny and Mineral Assessment, Institute of Mineral Resources, CAGS, 26 Baiwanzhuang Road, Beijing, 100037 China; 2 Graduate School of Oceanography, University of Rhode Island, Kingston, Rhode Island USA; 3 Chinese Academy of Geological Sciences, 26 Baiwanzhuang Road, Beijing, 100037 China

## Abstract

In classic orogenic models, the mountain range is underlain by a deep crustal root. Here we present the crustal and upper mantle structures along two receiver function profiles across Qilian, an orogen experiencing recent growth at the northern margin of the Tibetan plateau. Opposite to an expected crustal root beneath the orogen, the Moho beneath Qilian is arch-like, shallower beneath the center and deepens by up to 10 km beneath its southern and northern boundaries. Additional velocity interfaces sub-parallel to the Moho are observed in the lower crust of the basins south of Qilian, which we interpret as the top of a mechanically strong lower crust thrusting several tens of kilometers underneath Qilian. In the north, the small lateral offset between the surface and mantle traces of the thrust system reveals a steep boundary, indicating that the North China cratonic crust acts as a strong resistance to the northward growth of the plateau, forcing the development of the left-lateral strike-slip Haiyuan fault south of the northern Qilian suture. The young Qilian orogen thus has been rising and growing progressively from the boundaries to the center, squeezed up by more rigid tectonic blocks in the north and south.

## Introduction

Classic orogenic models are characterized by a deep crustal root^1^, which reflects a cumulative history of complex crustal shortening and folding, and subsequent surface erosion and crustal modification^2^. While the general mechanism of orogeny (crustal shortening) is widely agreed upon, details of the processes, through which orogenic belts develop their crustal roots, remain unclear.

**Figure 1:**
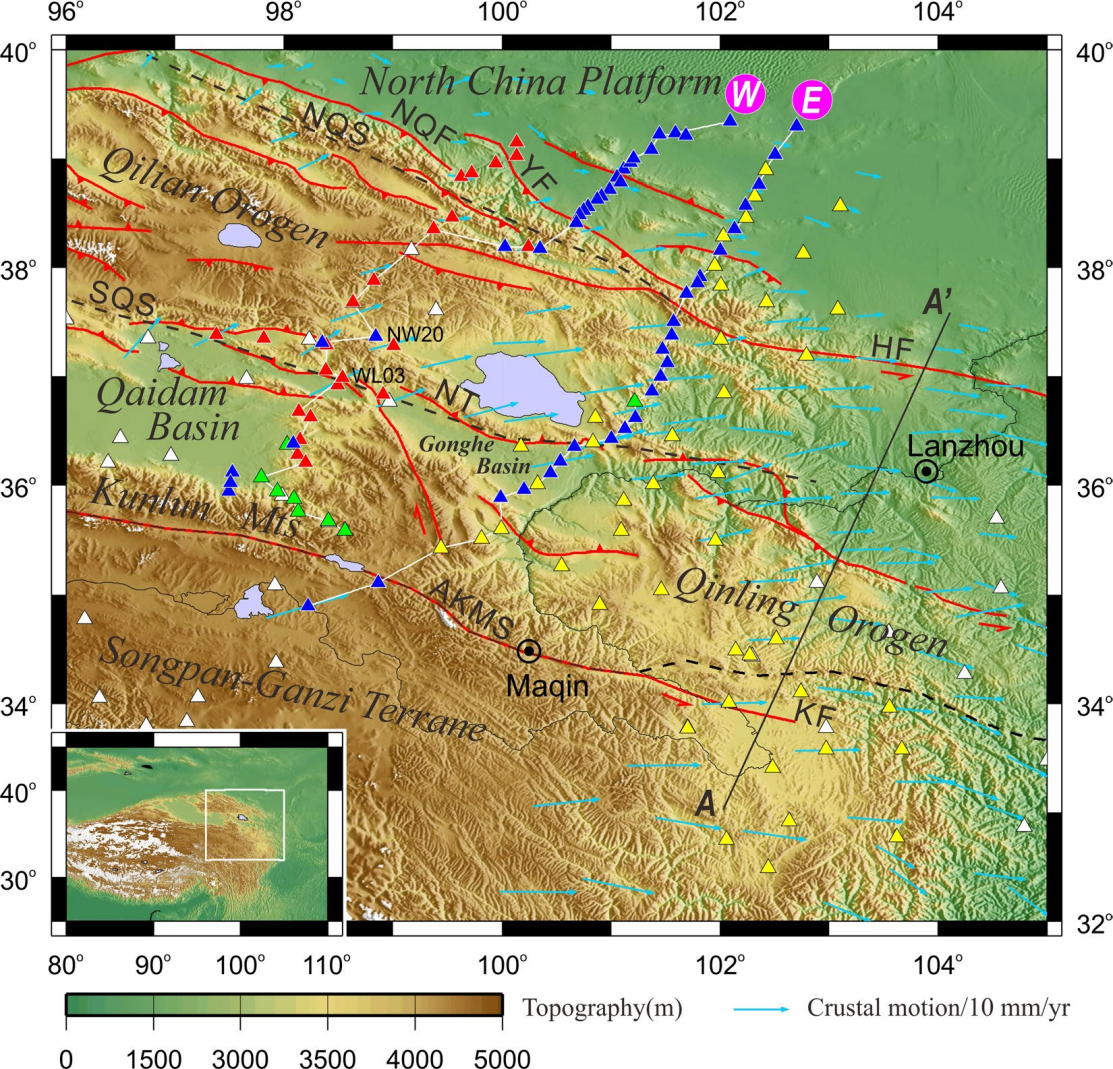
Location of the broadband seismic stations on the topographic map of the northeastern Tibetan plateau. Blue and red triangles are our seismic stations deployed in 2013 and in 2010^12^ respectively. Yellow and green triangles are our NETS seismic stations deployed in 2008 and in 2006 respectively^19^. White triangles show the ASCENT stations^13^. White lines connect stations used in the construction of the images in Figs 2 and 3, and the lettering ‘W’ and ‘E’ are the correspondent profiles (western and eastern lines). Dashed black lines mark the Anyimaqen-Kunlun-Muztagh (AKMS), Sourth Qilian (SQS) and North Qilian (NQS) sutures. Red lines delineate the major faults, including the left-lateral strike-slip Kunlun fault (KF), Qinghai Nanshan thrust (NT), North Qilian fault (NQF), Yumushan fault (YF) and Haiyuan fault (HF). Cyan arrows show the crustal motion relative to stable Eurasia observed by GPS^8^. Line AA’ denotes the profile by Ye *et al*. ^22^. Inset shows the extent of this map (white rectangle) within the plateau. Figure made with Generic Mapping Tools^37^ (GMT) v.4.2.0.

At the northeast margin of the Tibetan plateau, the Qilian terrane consists of complexly deformed earlier Paleozoic arcs^3, 4^ (Fig. 1). In the Carboniferous (359 to 299 million years ago), the earlier orogenic movement ceased and marine and marine-continental transition sedimentary layers covered the north Qilian^4^. The Cenozoic reactivation led to the rise of the present-day Qilian mountains, which are undergoing shortening and uplift^5–8^, driven presumably mainly by the collision of the Indian plate in the south and confined by the main Asian continental plate in the north. There are debates about whether the building of the Qilian mountains is driven also by southward subduction of the Asian mantle lithosphere^9–12^ or not^13, 14^. While controversy remains as whether deformation in the northeast Tibetan plateau started shortly after the collision of the Indian and Asian plates^6, 9^, there are mounting evidence showing that most of the orogenic growth in the region occurred since ~15 Ma^15, 16^. The present pattern of strike-slip dominated crustal deformation in the northeastern Tibetan plateau was established diachronously in late Miocene to Pliocene (11.6–2.6 Ma)^5, 17, 18^, making the present-day Qilian a relatively young orogen and a good place to understand early evolution of orogens.

## Data

In order to understand the ongoing crustal deformation of the northeast Tibetan plateau and its interaction with the main Asian continental plate, we deployed 47 broadband seismographs along two roughly linear profiles across the northeast Tibetan plateau between 2013 and 2014 (Fig. 1). We used the seismic data from these 47 stations and 33 previous stations in the vicinity of our profiles in this study. The stations were equipped with Guralp 3ESP or 3T broadband seismometers. Both profiles are about 500 km long, running in the SSW–NNE directions. The western profile starts from just north of the Kunlun fault (KF), across the eastern Qaidam basin and the Qilian orogen into the North China platform (NCP, also known as the Gobi Alashan block), with its southern part following our pilot profile of the joint Sino-US North-Eastern Tibetan plateau Seismic (NETS) stations deployed between summers of 2006 and 2007^19^ and the northern part following the Chinese Geological Survey (CGS) stations deployed between the summers of 2010 and 2011^12^. The eastern profile extends from the northern margin of the Songpan-Ganzi terrane, across the KF, the Gonghe basin and the Qilian orogen to the NCP. The eastern profile follows the western flank of the NETS areal stations deployed from 2008 to 2009. Both profiles trend more or less perpendicular to the mountain ranges and the main tectonic boundaries in the region, including the left-lateral Kunlun strike-slip fault, the South Qilian suture (SQS), the North Qilian suture (NQS), and Yumushan fault (YF). These two linear profiles are designed for cross-section imaging of the crustal and upper mantle structure with receiver function profiling techniques (see Method).

## Results

**Figure 2:**
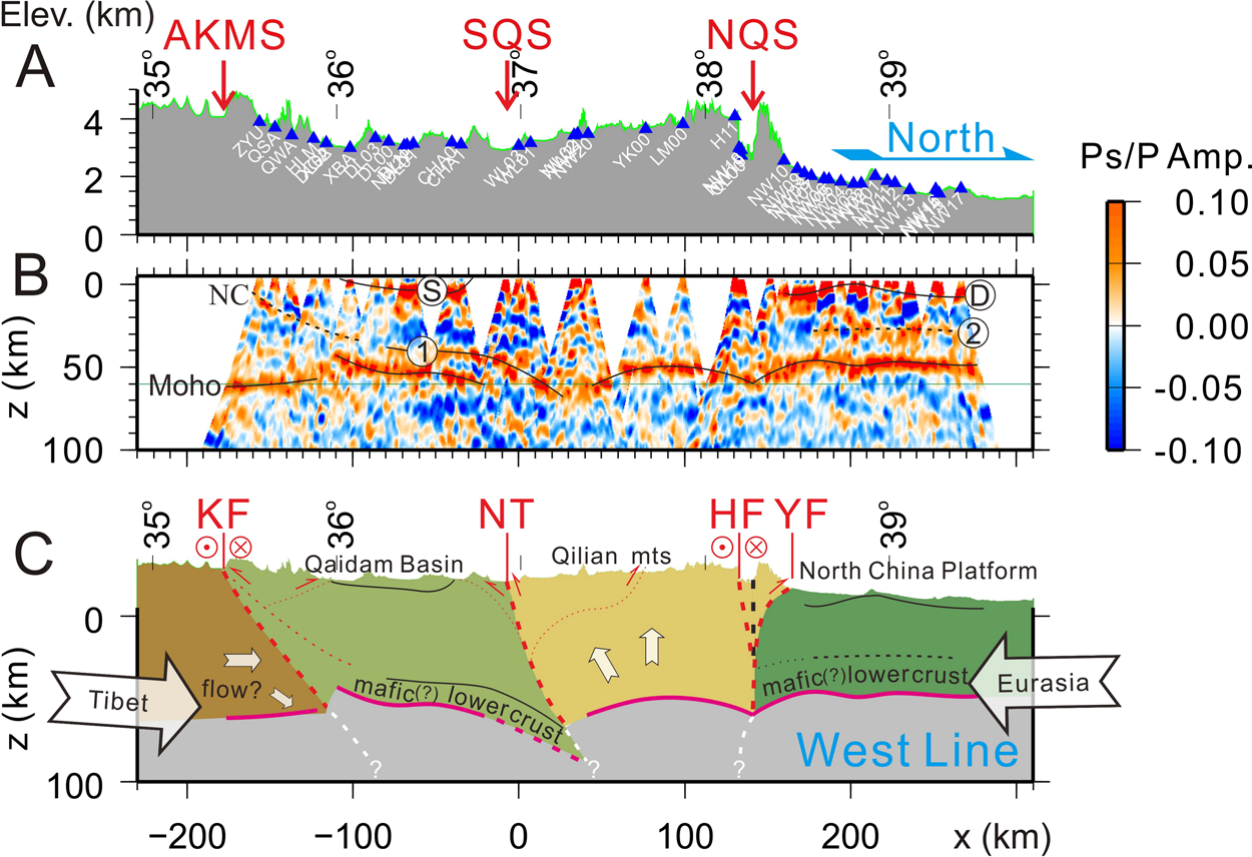
Seismic image and interpretations of crustal structure along the western profile. (**A**) Topography and station positions along the profile. (**B**) Seismic cross-section constructed with 4005 P-wave receiver functions along the profile. Positive and negative amplitudes are plotted in red and blue, respectively marking interfaces with increasing and decreasing impedance with depth. Horizontal distances are referenced to the station WL03 (98.53548°E, 36.98834°N) near the SQS, and vertical distances are relative to sea level. Thin green line at 60 km depth is plotted for reference only. (**C**) Interpretive cross-sections of the northeastern Tibetan plateau along the western profile. Mechanically strong (mafic?) rocks are inferred in the lower crust of the Qaidam basin (between ‘1’ and Moho), and the middle to lower crust of the cratonic North China platform (between ‘2’ and Moho). Solid and dashed lines denote observed and inferred interfaces.

**Figure 3:**
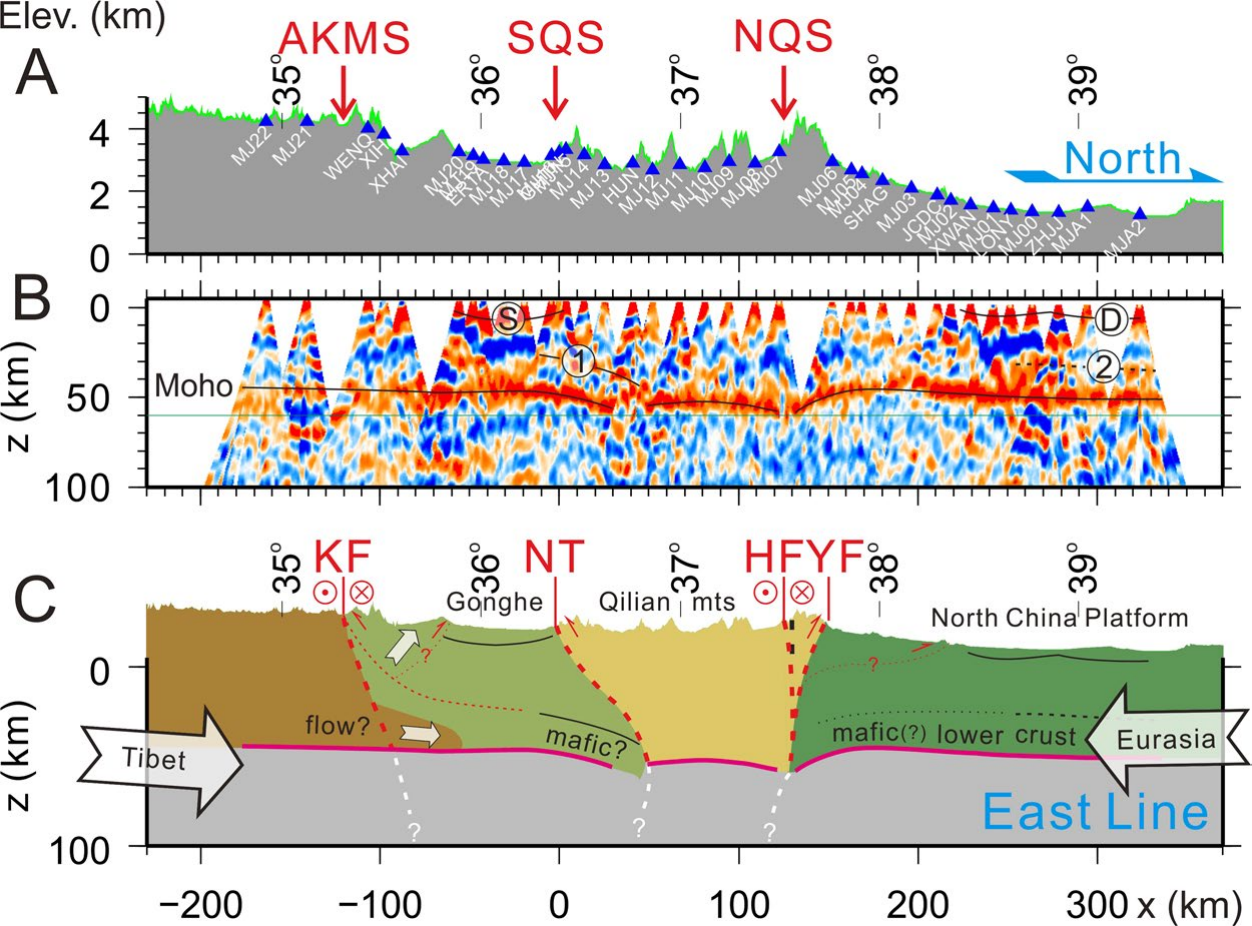
Seismic image and interpretations of crustal structure along the eastern profile. (**A**) Topography and station positions along the profile. (**B**) Seismic cross-section constructed with 3693 P-wave receiver functions along the profile. Horizontal distances are referenced to the station GHTN (100.83083°E, 36.3925°N) near the SQS, and vertical distances are relative to sea level. Color scale is the same as in Fig. 2. (**C**) Interpretive cross-sections of the northeastern Tibetan plateau along the western profile. Other annotation as in Fig. 2.

**Figure 4:**
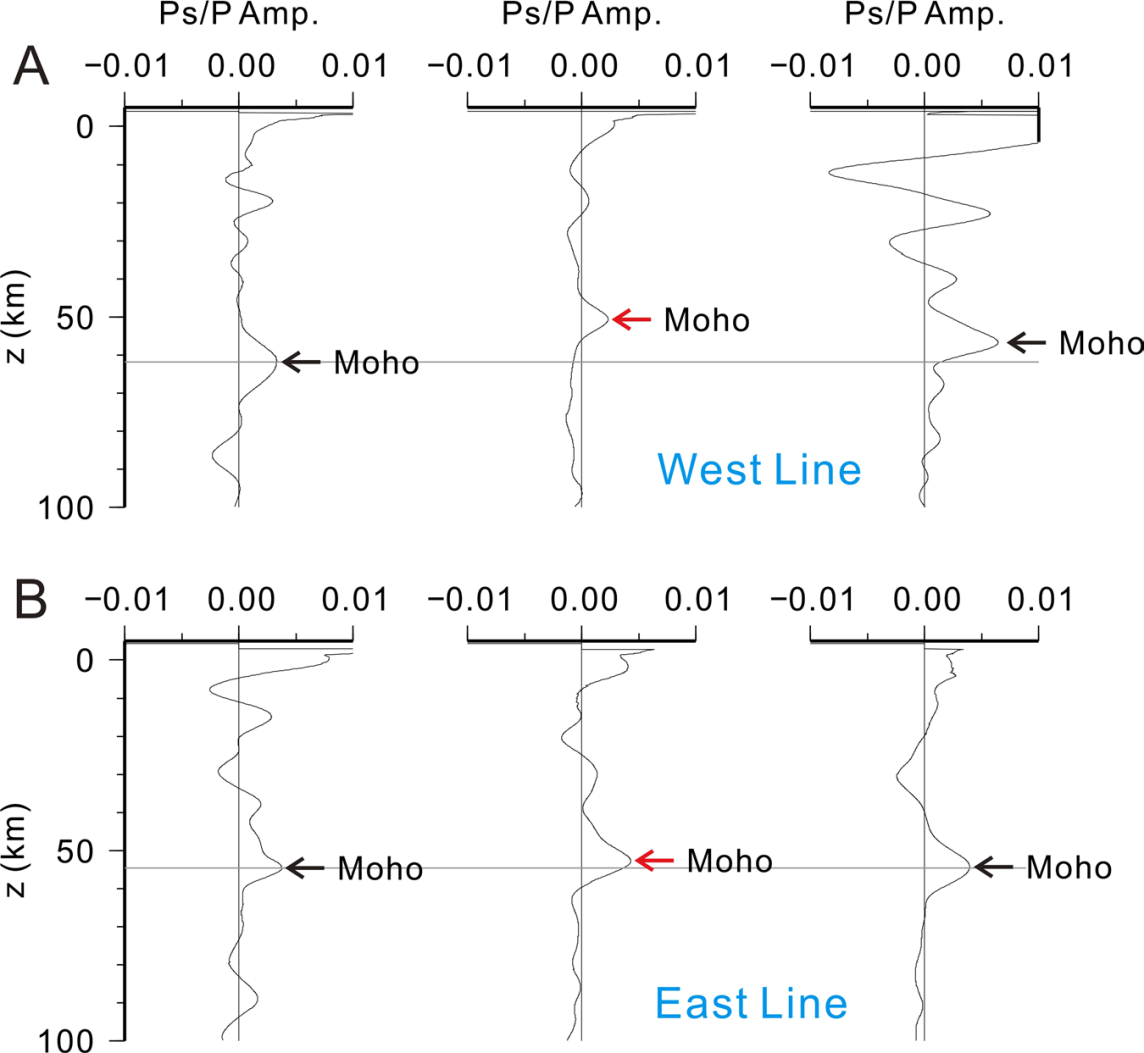
Stacked receiver functions along sections of the western and eastern profiles. (**A**) From left to right, stacked receive functions for the southern (x = 40–60 km in Fig. 2), middle (x = 70–110) and northern (x = 120–140) sections of the western profile within the Qilian orogen. (**B**) Similarly for the eastern profile: the southern (x = 30–50 km in Fig. 3), middle (x = 70–90) and northern (x = 100–120) sections.

Our two receiver function profiles (Figs 2 and 3) reveal a crustal structure beneath Qilian that is opposite to the expected deep crustal root in classic orogenic models^1, 20, 21^. At about 50~58 km below the sea level (BSL), the Moho beneath Qilian is arch-like, more obvious on the western profile, deepening from ~50 km BSL at the center to 55–60 km BSL at the northern and southern boundaries of the orogen, respectively (Fig. 2, Figs S3 and S5). To highlight this arch-like feature, we split the southern, middle and northern sections of the Qilian orogen and stacked the receiver functions within each section to enhance the signal of the Moho. Figure 4 shows that the Moho beneath the center of the orogen is unequivocally shallower than its northern and southern sections along the western profile. On the eastern profile, the Moho arch is less pronounced, deepening by about 3 km from the center of the Qilian orogen to the boundaries (Figs 3 and 4 and Fig. S4). Along a similar receiver function profile ~200 km east of our eastern profile (AA’ in Fig. 1), the Moho under the less-developed east Qilian orogen is nearly flat at ~48 km BSL^22^.

There are major differences in the way the Qilian crust interacts with the tectonic blocks north and south of the orogen. In the north, the Moho beneath the NCP is characterized by consistently high amplitudes of the converted seismic waves on the two profiles (Figs 2 and 3). The small perturbations to the Moho near ~39°N on the western profile and near 38.5°N on the eastern profile can be attributed to multiple waves converted and reflected at the base of sedimentary rocks (marked ‘D’ beneath the NCP in Figs 2 and 3). The slight northward deepening of the Moho at the northern ends of the two profiles is most likely due to the under-corrected effects of the increasingly thick sedimentary layer toward north, because the shallow velocity near the northern ends of the profiles is not well constrained due to a lack of seismic stations there^23^. The Moho beneath the NCP remains relatively flat, at about ~48 km BSL on the western profile and ~45 km BSL on the eastern profile. It deepens south of the YF and reaches ~58 km BSL beneath the NQS. The left-lateral strike-slip Haiyuan fault, which accommodates the eastward motion of the crust in the northeastern Tibetan plateau, is tens of kilometers south of the NQS near the western profile and merges with the NQS near the eastern profile (Fig. 1). Beneath both profiles, the most depressed points of the Moho near the southern end of the NCP section are directly beneath the surface trace of the NQS (Figs 2, 3 and Fig. S5). The lateral distance between the surface trace of the thrust fault and the most depressed points of the Moho are about 25 km on the two profiles, suggesting a steep thrust system between the Qilian orogen and the NCP, in contrast to the shallow underthrust at the southern margin of the Tibetan plateau^24^. This observation supports the notion of southward under-thrusting of the NCP crust^11^, at least at the crustal level, as under-thrusting of the crust and mantle lithosphere places the southern edge of the NCP Moho south of the surface trace of the thrust fault, as observed.

South of the Qilian orogen, the Qaidam Moho is seen clearly deepening to north from ~48 km BSL north of the Kunlun mountains to ~63 km BSL beneath the SQS on the western profile (Fig. 2 and Fig. S3). From this point to farther north, the Moho becomes less certain to discern on this profile, but for reasons given below, we infer that the Qaidam Moho extends north of the surface trace of the SQS. In contrast, the corresponding Moho on the eastern profile is nearly horizontal south of 36°N, at ~50 km BSL (Fig. 3). Farther north, it deepens slightly, to ~57 km BSL, and can be traced continuously to ~30 km north of the SQS (Fig. 3). The Moho is somewhat blurred beneath the Gonghe basin (~35.7–36.3°N) on the eastern profile, probably due to multiple waves converted and reflected at the bottom of the Gonghe basin since the directly converted-wave (marked ‘S’ beneath the Gonghe basin in Fig. 3) is very strong and clearly seen on the profile.

At ~15–20 km above the Moho south and near the SQS, there is a prominent phase corresponding to a positive velocity contrast interface (increasing velocity with depth) at ~40–60 km BSL on the western profile and at ~30–40 km BSL on the eastern profile (marked ‘1’ in Figs 2 and 3). The southward extension of this velocity interface is obscured by the multiple waves from the basin sedimentary layer. Feng *et al*. (2014) also observed such an interface^12^ nearly along our western profile and stressed its similarity with the doublet phase observed beneath southern Tibet^11, 25, 26^. The doublet phase beneath southern Tibet appears more or less horizontal within a narrow depth range of ~55–58 km BSL, consistent with the interpretation of an eclogitization phase change boundary^11, 26, 27^. In contrast, the lower crustal velocity interface beneath the Qaidam and Gonghe basins runs parallel to the Moho and is clearly not horizontal (thus at very different confining pressures). If this interface is the eclogite phase boundary, the deepening interface requires a substantially colder lower crust at a greater depth towards the SQS, an unlikely circumstance. We thus interpret this lower crustal velocity interface as the top of the lower crust, which is likely mafic to have the high velocity needed to create a large enough velocity contrast with the crust above. In either case, the Moho, the bottom of this lower crust or an unlikely eclogitized lower crust, must follow the top interface to 20–40 km north of the SQS beneath the western profile (Fig. 2), similar to the Moho on the eastern profile (Fig. 3). A possible explanation for the lack of a clear arrival from the Moho north of the SQS on the western profile is that a strong lower crustal layer at the bottom of the Qaidam crust at greater depths may significantly reduce the impedance contrast of this part of the Moho, therefore reducing the amplitude of the converted seismic waves and making this part of the Moho more difficult to identify.

## Discussion

The western Qilian terrane appears to be a mechanically weak and recently reactivated orogen bounded by two mechanically strong tectonic blocks: The NCP and Qaidam basin. The west Qilian is characterized by a low velocity zone in its mid-to-lower crust and its Vp/Vs ratio is typical for more felsic crustal composition^23, 28^. In the north, the Qilian crust abuts the western block of the North China craton consisting of a basement of Precambrian metamorphic rocks^29^, which is generally considered to be older, colder and mechanically stronger than the Qilian’s basement that experienced oceanic subduction and arc magmatism between 520–400 Ma^3^. GPS data show that the strain rate within the Qilian orogen is an order of magnitude greater than the rate within the western block of the north China craton^7, 8^. In the south, the basement of the Qaidam basin is composed of Precambrian-Silurian ( >419 Ma) metamorphic rocks^30^. The Qaidam crust and the mantle lithosphere appears to be exceptionally strong mechanically, with an effective elastic thickness of ~70 km, significantly greater than that of 10–30 km for the rest of the Tibetan plateau^31^. Seismic studies show that the lower crust beneath the Qaidam and Gonghe basins has a much higher velocity than in the surrounding tectonic units^23^, also indicating a mechanically strong lower crust.

Taken together, the receiver function profiles suggest that the recent thickening of the Qilian orogenic crust is most developed at its northern and southern boundaries, squeezed up by under-thrusting of a mechanically strong Qaidam crust in the south and blocked by the North China cratonic crust in the north. Analysis of surface geology and reconstruction of seismic reflection profiles reveal that the shortening along the northern Qilian Shan frontal thrust system is much higher than in the interior of Qilian^32^, consistent with our observation of a thicker crust near the northern boundary. The arch-like Moho across Qilian may characterize crustal deformation of young orogens sandwiched between strong tectonic blocks. The surface topography and Moho display mirror images, particularly on the eastern profile (Fig. 3), reflecting a component of the “pure-shear” mode of crustal deformation^33^ on the cross-sections, in a region otherwise dominated by strike-slip crustal deformation^34^.

The development of the Moho arch appears to be correlated with the development of the orogen from the planar Moho under the less-developed eastern Qilian orogen 200 km east of our eastern profile^22^, to the small Moho arch under our eastern profile (Figs 3 and 4) and finally the most pronounced Moho arch beneath the more developed western Qilian (Figs 2 and 4). We speculate that as the tectonic blocks continue to converge, crustal thickening will propagate from the boundaries to the center and the arch-like Moho will eventually disappear, forming a classic orogenic crust root beneath the orogen.

## Methods

In this study, we obtained the images of the crustal and upper-mantle structure of the NE Tibetan plateau using common-conversion-point (CCP) stacks of P-wave receiver functions (RFs), a well-established method that has been applied in many similar studies of the Tibetan plateau^11, 26^. This method enhances converted S waves produced by P waves of distant earthquakes impinging on seismic interfaces. The interfaces and their properties can be determined from the converted waves based on the time delays between the direct and converted waves, which are mainly proportional to the depths of the interfaces, and on the amplitudes of the converted waves, which depend on the magnitudes and signs of the velocity contrasts.

We used teleseismic events with magnitudes greater than 4.5 and epicentral distances greater than 30°. Most of them occurred to the east of the profiles (Fig. S1). To ensure that only good-quality waveforms are used, we also applied a visual selection process to all the data. We performed time-domain iterative deconvolution^35^ in the calculation of RFs, in which the Gaussian filter factor was set to 2.5 to retain sufficient high frequency (up to 1 Hz) signals to image the crustal and upper-mantle structure. We isolated the converted waves from the incident waves using the wave-vector method^36^ which decomposes P- and S-waves and removes the effect of the free surface by multiplying the inverse matrix of free-surface response with the observed vertical, radial and transverse components of data. A free-surface response matrix is calculated for surface velocities of Vp = 5.34 km/s and Vs = 3.00 km/s, which removed most of the effect of the free surface. We used variable reference models for the individual stations (Fig. S2) to migrate the RFs of different seismic stations from time to space in a 3-D volume by tracing the rays from the location of each seismic station. The reference models were obtained by interpolations at each station of the shear-wave velocity model derived from joint inversion of receiver functions and Rayleigh wave dispersion^23^, and the Vp/Vs ratios derived from h-k stacking of RFs obtained previously^23^ and in this study (Table S1). Then all the RFs were projected and stacked onto cross-sections aligned to due north with a fixed horizontal stacking bin width of 1 km.

### Data Availability

The original seismic data from the NETS and ASCENT experiments are available at the IRIS DMC. Receiver functions from all the stations used in this study, including non-IRIS data, are available upon request from D.S.

## Electronic supplementary material

Supplementary Information

## References

[1] Dewey JF, Bird JM (1970). Mountain belts and the new global tectonics. J. Geophys. Res..

[2] Fischer KM (2002). Waning buoyancy in the crustal roots of old mountains. Nature.

[3] Yin A (2007). Early Paleozoic Tectonic and Thermomechanical Evolution of Ultrahigh-Pressure (UHP) Metamorphic Rocks in the Northern Tibetan Plateau, Northwest China. International Geology Review.

[4] Song S, Niu Y, Su L, Xia X (2013). Tectonics of the north Qilian orogen, NW China. Gondwana Res..

[5] Chen Z (2000). Global positioning system measurements from eastern Tibet and their implications for India/Eurasia intercontinental deformation. J. Geophys. Res..

[6] Yin A, Harrison TM (2000). Geologic evolution of the Himalayan–Tibetan orogen. Annu. Rev. Earth Planet. Sci..

[7] Wang Q (2001). Present-day crustal deformation in China constrained by global positioning system measurements. Science.

[8] Zhang PZ (2004). Continuous deformation of the Tibetan plateau from global positioning system data. Geology.

[9] Meyer B (1998). Crustal thickening in Gausu-Qinghai, lithospheric mantle subduction, and oblique, strike-slip controlled growth of the Tibet plateau. Geophys. J. Int..

[10] Tapponnier P (2001). Oblique stepwise rise and growth of the Tibet Plateau. Science.

[11] Kind R (2002). Seismic images of crust and upper mantle beneath Tibet: Evidence for Eurasian plate subduction. Science.

[12] Feng M (2014). Structure of the crust and mantle down to 700km depth beneath the East Qaidam basin and Qilian Shan from P and S receiver functions. Geophys. J. Int..

[13] Yue H (2012). Lithospheric and upper mantle structure of the northeastern Tibetan Plateau. J. Geophys. Res..

[14] Liang X (2012). A complex Tibetan upper mantle: a fragmented Indian slab and no south-verging subduction of Eurasian lithosphere. Earth Planet. Sci. Lett..

[15] Fang X, Li J, Song C, Yan M (2013). Late Miocene-Quaternary synchronous-but-magnitude-different episodic rapid uplifts of the NE Tibetan plateau: A synthesis from flexural basins. ACTA Geol. Sinica (English ed.).

[16] Yuan D-Y (2013). The growth of northeastern Tibet and its relevance to large-scale continental geodynamics: A review of recent studies. Tectonics.

[17] Tapponnier P (1990). Active thrusting and folding in the Qilian Shan, and decoupling between upper crust and mantle in northeastern Tibet. Earth Planet. Sci. Lett..

[18] Zheng D, Clark MK, Zhang P, Zheng W, Farley KA (2010). Erosion, fault initiation and topographic growth of the North Qilian Shan (northern Tibetan Plateau). Geosphere.

[19] Shi D, Shen Y, Zhao W, Li A (2009). Seismic evidence for a Moho offset and south-directed thrust at the easternmost Qaidam–Kunlun boundary in the Northeast Tibetan plateau. Earth planet. Sci. Lett..

[20] Oxburgh ER (1969). The deep structure of orogenic belts - the root problem. Geological Society, London, Special publications.

[21] Vanderhaeghe O, Medvedev S, Fullsack P, Beaumont C, Jamieson RA (2003). Evolution of orogenic wedges and continental plateau: insights from crustal thermal-mechanical models overlying subducting mantle lithosphere. Geophys. J. Int..

[22] Ye Z (2015). Seismic evidence for the North China plate underthrusting beneath northeastern Tibet and its implications for plateau growth. Earth Planet. Sci. Lett..

[23] Zheng D (2016). Crustal and upper mantle structure beneath the northeastern Tibetan Plateau by joint analysis of receiver functions and Rayleigh wave dispersion. Geophys. J. Int..

[24] Schulte-Pelkum V (2005). Imaging the Indian subcontinent beneath the Himalaya. Nature.

[25] Yuan X, Ni J, Kind R, Mechie J, Sandvol E (1997). Lithospheric and upper mantle structure of southern Tibet from a seismological passive source experiment. J. Geophys. Res..

[26] Nábělek J (2009). Underplating in the Himalaya-Tibet collision zone revealed by the Hi-CLIMB experiment. Science.

[27] Shi D (2015). Receiver Function Imaging of Crustal Suture, Steep Subduction, and Mantle Wedge in the Eastern India-Tibet Continental Collision Zone. Earth Planet. Sci. Lett..

[28] Li H (2014). The distribution of the mid-to-lower crustal low-velocity zone beneath the northeastern Tibetan Plateau revealed from ambient noise tomography. J. Geophys. Res. Solid Earth.

[29] Vincent SJ, Allen MB (1999). Evolution of the Minle and Chaoshui Basins, China: Implications for Mesozoic strike-slip basin formation in central Asia. Geol. Soc. Am. Bull..

[30] Wu, C., Yin, A., Zhang, J.Y., Liu, W.C. & Ding, L. Geologic history of the Paleozoic-Mesozoic Eastern Kunlun Arc, central Tibetan Plateau: Implications for the tectonic evolution of the Tethyan orogenic system. *Lithosphere*, in press.

[31] Braitenberg C, Wang Y, Fang J, Hsu HT (2003). Spatial variations of flexure parameters over the Tibet-Qinghai plateau. Earth Planet. Sci. Lett..

[32] Zuza, A.V., Cheng, X. & Yin, A. Testing models of Tibetan plateau formation with Cenozoic shortening estimates across the Qilian Shan-Nan Shan thrust belt. Geosphere **12** (2), doi:10.1130/GES01254.1 (2016).

[33] England PC, Houseman GA (1986). Finite strain calculations of continental deformation, 2, Comparison with the India-Asia collision zone. J. Geophys. Res..

[34] Tapponnier, P., Peltzer, G., Le Dain, A.Y., Armijo, R. & Cobbold, P. Propagating extrusion tectonics in Asia: New insights from simple experiments with plasticine. *Geology* **10**, 611–616, doi:10.1130/0091-7613(1982) 10<611:PETIAN>2.0.CO;2 (1982).

[35] Ligorria, J. & Ammon, C.J. Iterative deconvolution and receiver function estimation. *Bull. Seismol. Soc. Am*. **89**, 1395–1400, doi:10.1.1.473.9379 (1999).

[36] Reading A, Kennett B, Sambridge M (2003). Improved inversion for seismic structure using transformed, S-wave vector receiver functions: Removing the effect of the free surface. Geophys. Res. Lett..

[37] Wessel P, Smith WHF (1998). New, improved version of generic mapping tools released. EOS Trans..

