# Supplementary material for: Growth of the northeastern margin of the Tibetan Plateau by squeezing up of the crust at the boundaries
Source: Sci Rep. 2017 Sep 6;7:10591. doi: 10.1038/s41598-017-09640-0 (PMC5587744; doi:10.1038/s41598-017-09640-0)
Supplement: Supplementary file 1 — Supplementary Information [file 41598_2017_9640_MOESM1_ESM.doc]

Growth of the northeastern margin of the Tibetan Plateau by squeezing up of the crust at the boundaries

**Jianyu Shi ^1^, Danian Shi ^1^, Yang Shen ^2,*^, Wenjin Zhao ^3^, , Guangqi Xue ^1^, Heping Su ^1^, and Yang Song ^3^**

*^1^MLR Key Laboratory of Metallogeny and Mineral Assessment, Institute of Mineral Resources, CAGS, 26 Baiwanzhuang Road, Beijing 100037, China*

*^2^Graduate School of Oceanography, University of Rhode Island, Kingston, Rhode Island, USA*

*^3^Chinese Academy of Geological Sciences, 26 Baiwanzhuang Road, Beijing 100037, China*

**
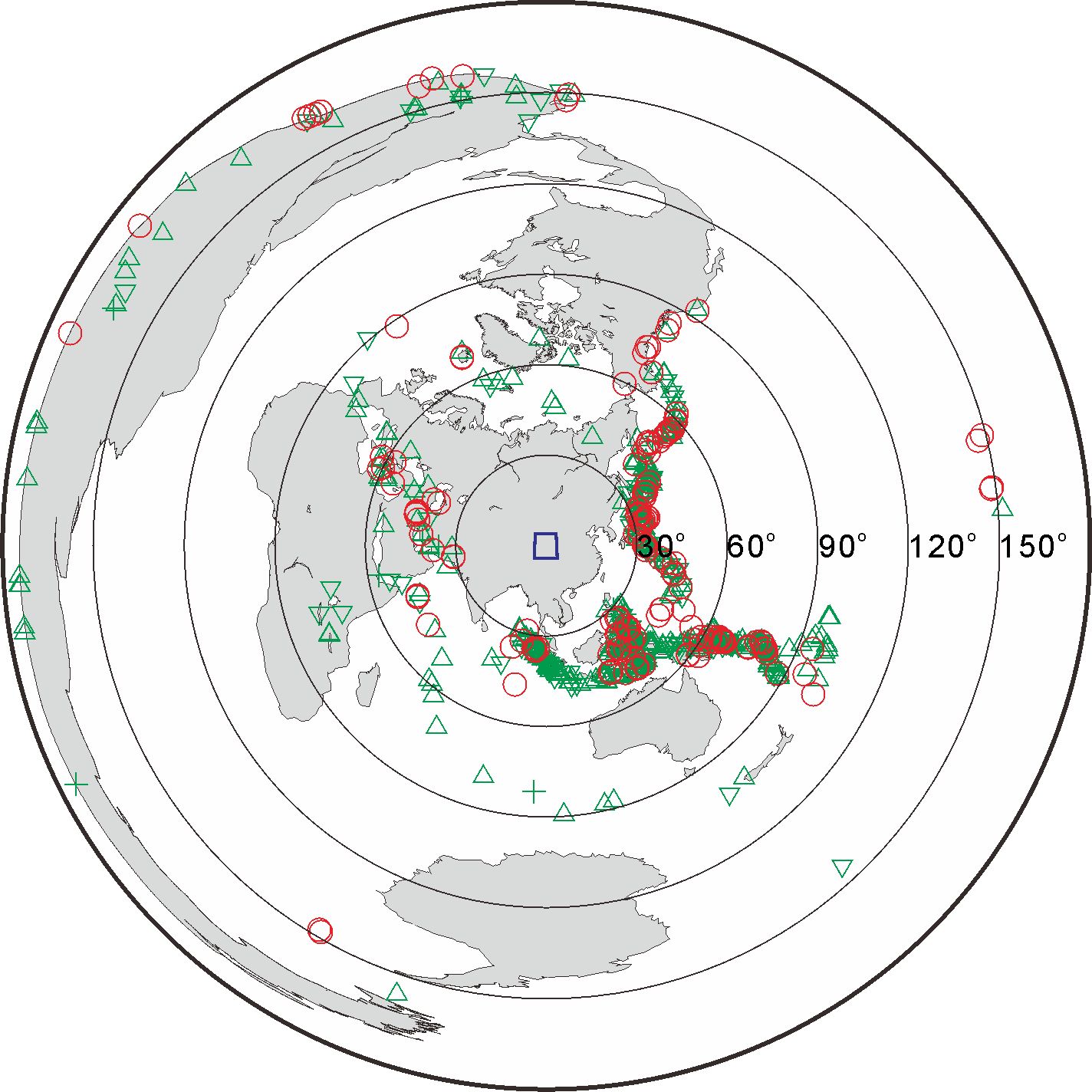
**

**Figure S1. Distribution of earthquakes used to construct receiver function images**. Red circle, green cross, normal and inverted triangle indicate the earthquakes observed by stations deployed in 2013, 2010, 2008 and 2006 respectively. The map is centered at the center of the study region (100.5°E, 36°N) and a blue rectangle denotes the location of Fig. 1. The Generic Mapping Tool^37^ (GMT v.4.2.0) and the earthquake locations in the U.S. Geological Survey earthquake catalog (URL: <https://earthquake.usgs.gov/earthquakes/search/>, accessed June 10, 2015) were used to generate this figure.


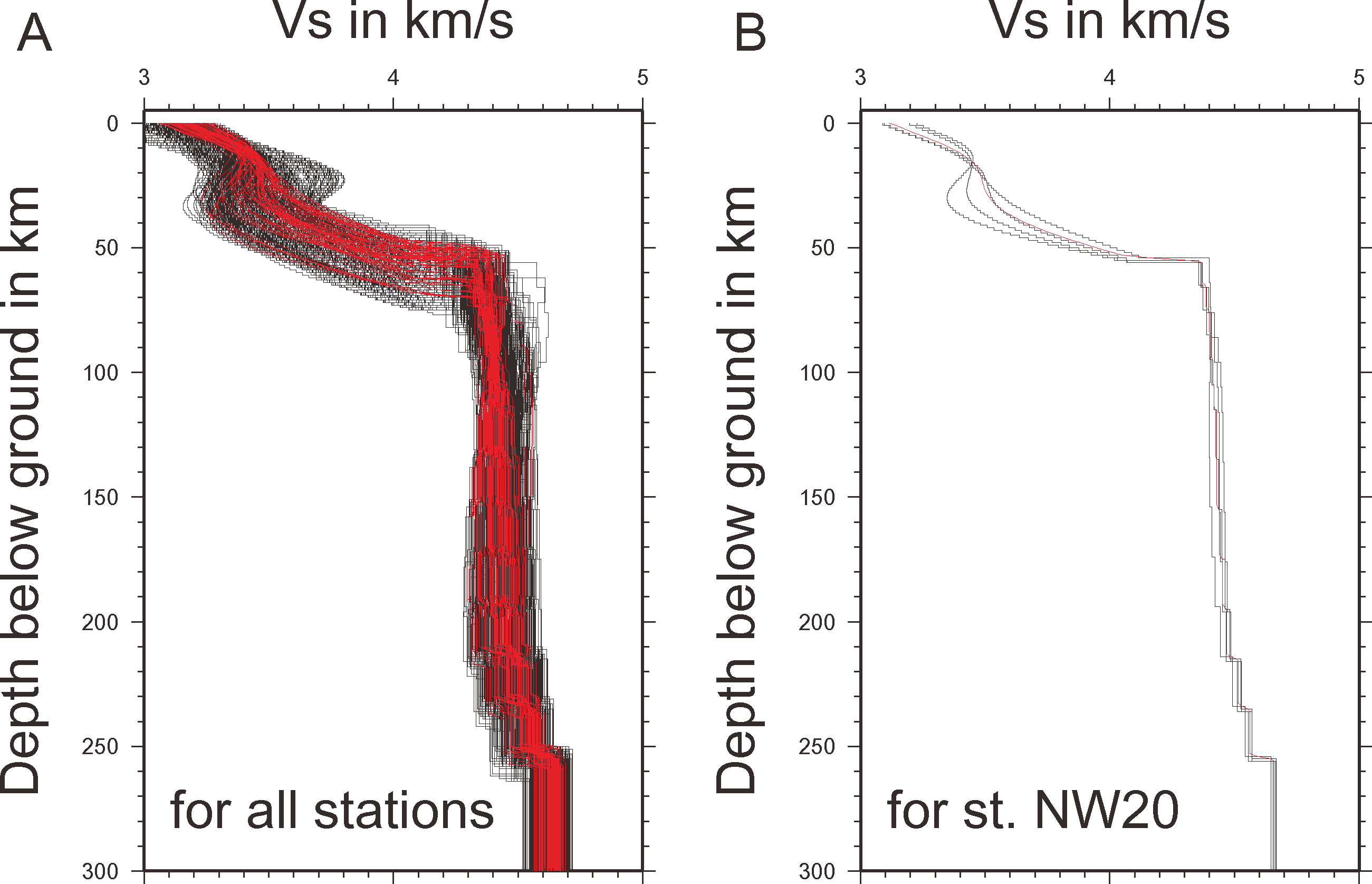


**Figure S2. The velocity model used to migrate the receiver functions for all the stations (A) and an example station NW20 (B)**. Black curves are the velocities at all the 0.5°×0.5° grid intervals from joint inversion of receiver functions and Rayleigh wave dispersions**^23^**. Red curves are those interpolated at each station from 4 neighboring grids surrounding the station.

**
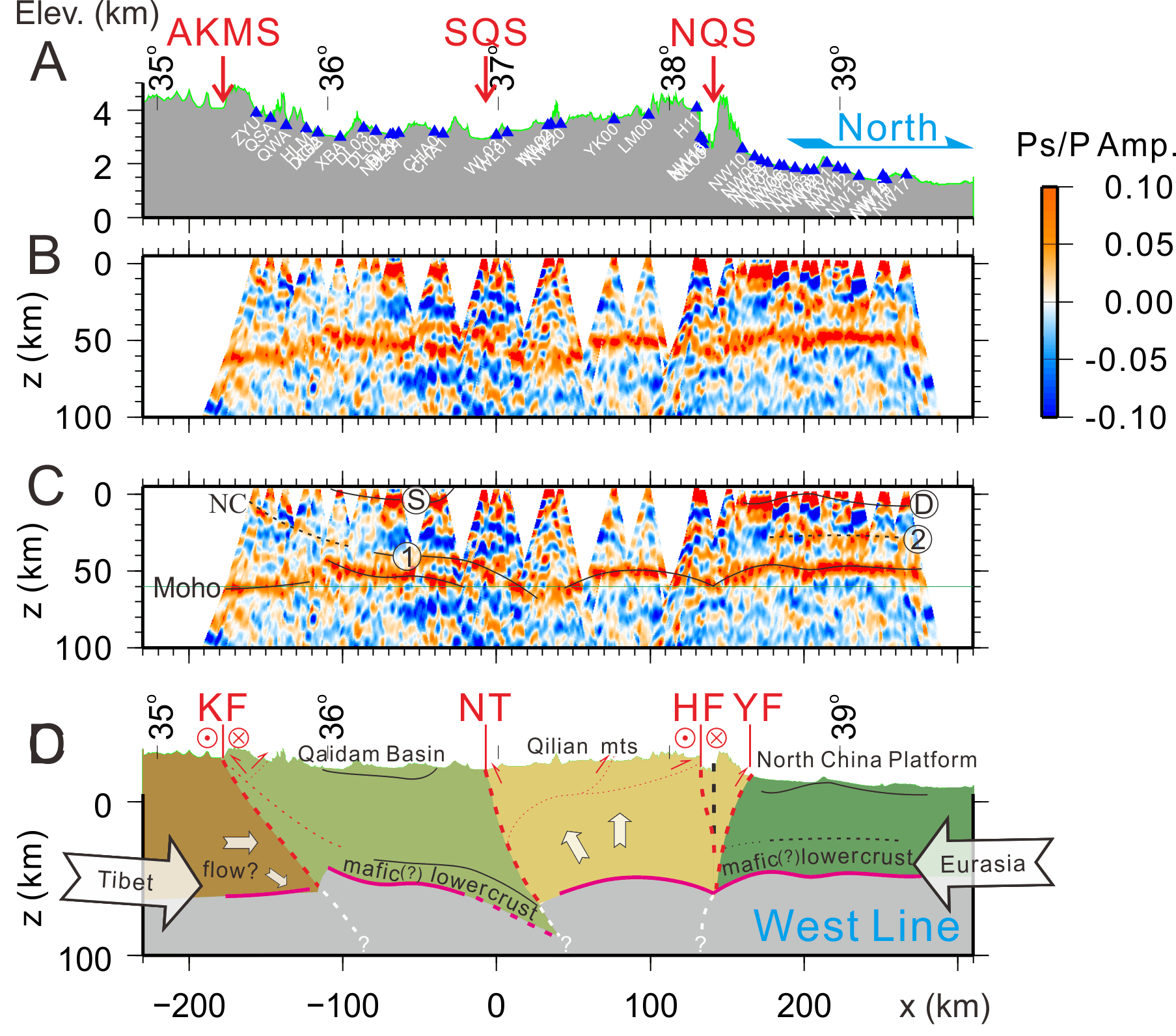
**

**Figure S3. The same data profile of Fig. 2 but also including the seismic cross-section without any interpretations (B).**


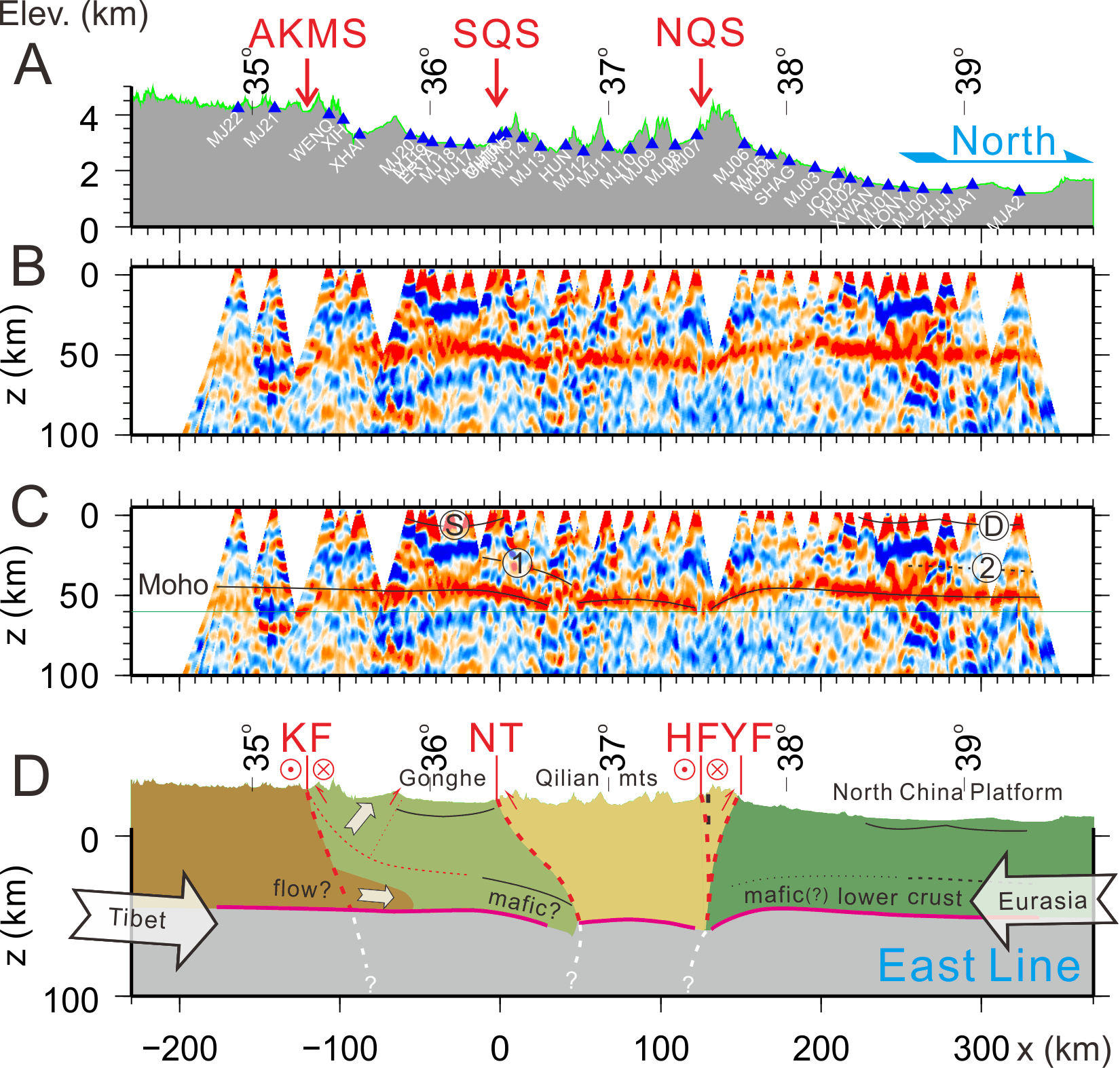


**Figure S4. The same data profile of Fig. 3 but also including the seismic cross-section without any interpretations (B).**


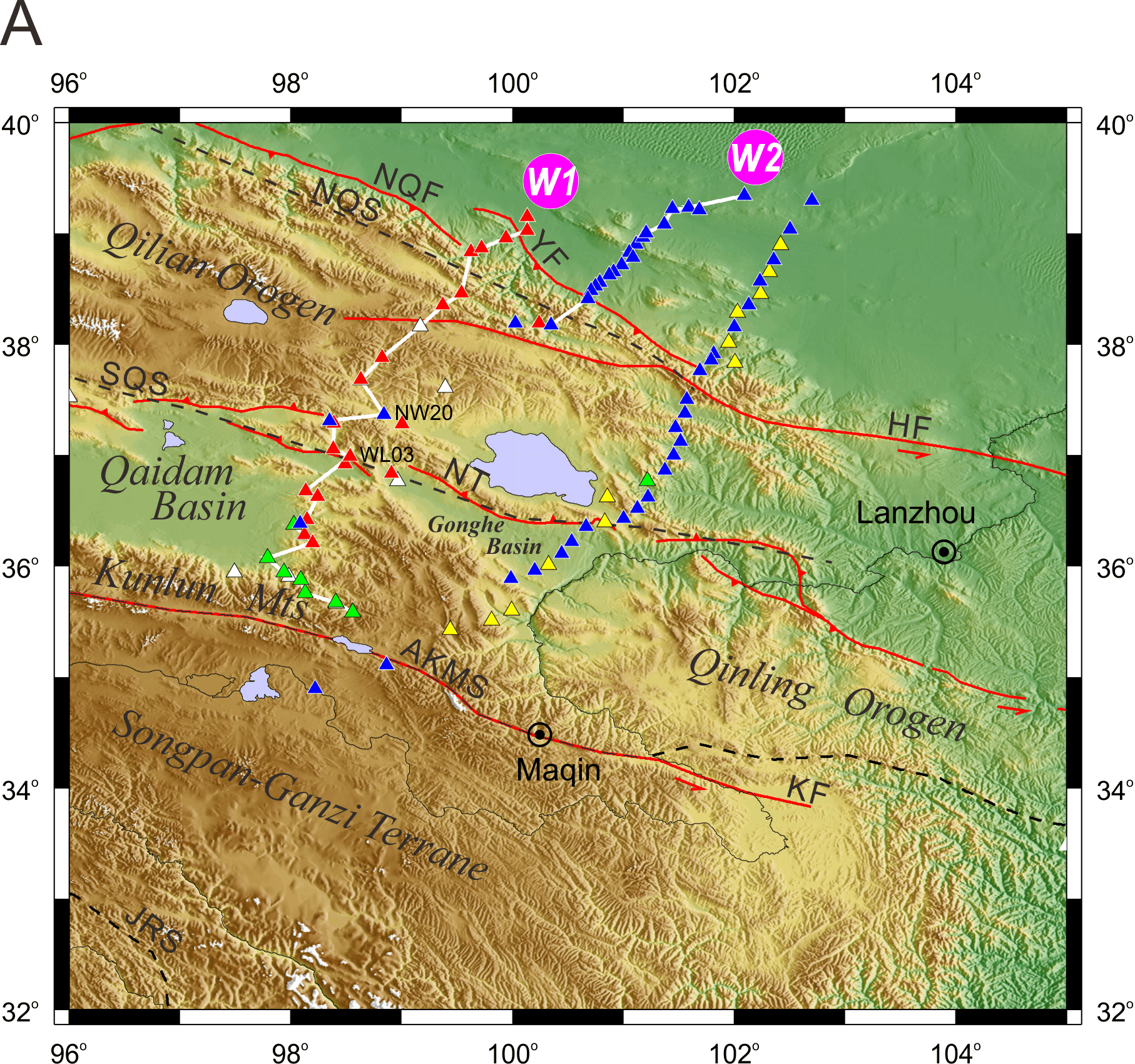


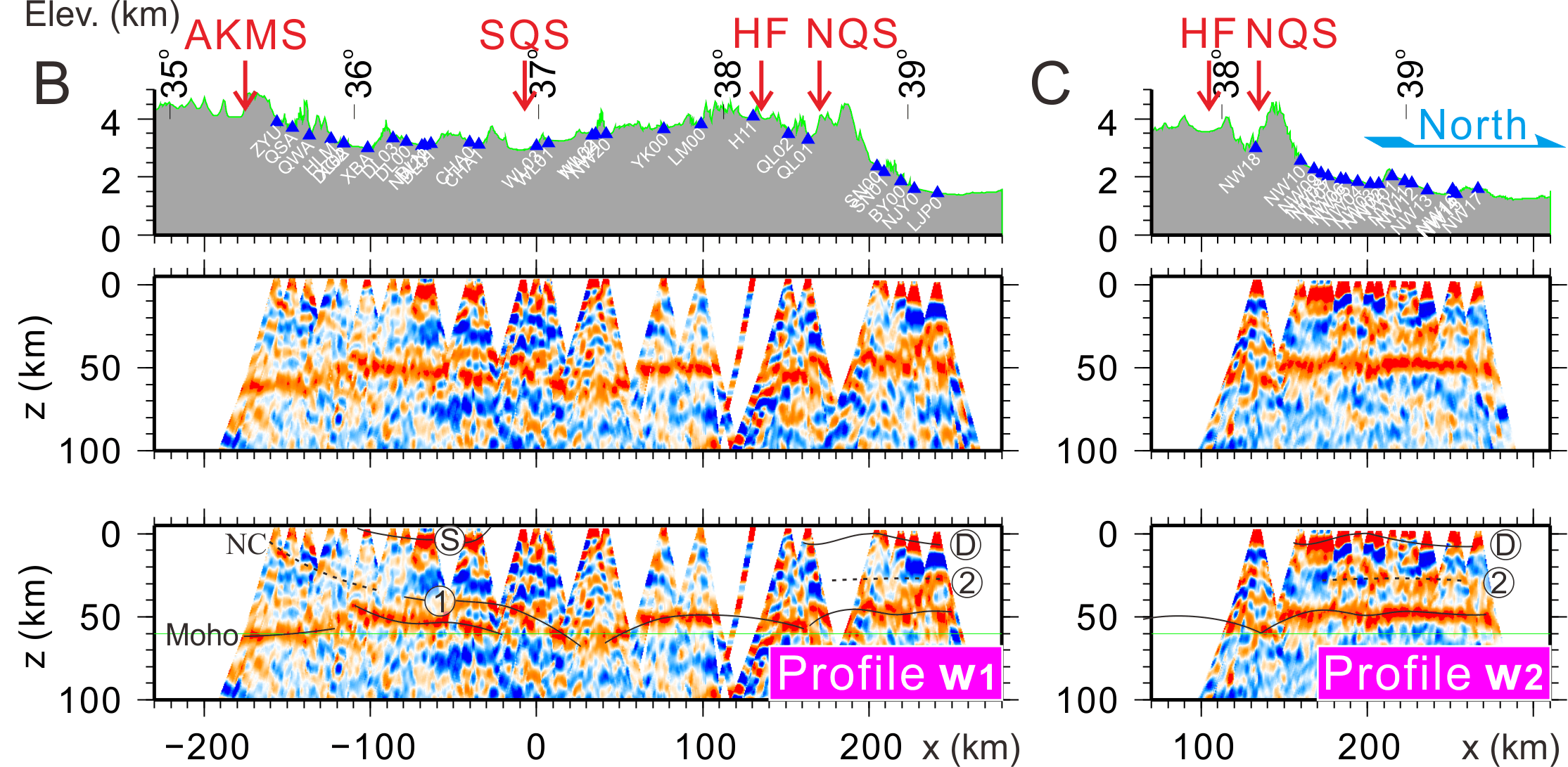


**Figure S5. The same data profile of Fig. 2 but split into two separated cross-sections w1 and w2, showing the most depressed points of the Moho are more closely correlated to the NQS than the HY.** (A) Shows the locations of the two cross-sections. (B) and (C) The topography, seismic images and interpretations along the cross-sections w1 and w2 respectively. Figure made with Generic Mapping Tools^37^ (GMT) v.4.2.0.

**Table S1.** Vp/Vs ratios used in the migration from time to depth in the common conversion point (CCP) stack of receiver functions for the crustal structure

| **Station** | **Latitude**  **(°N)** | **Longitude**  **(°E)** | **Altitude**  **(m)** | **Dist. from NQS (km)** | **Moho**  **(km)** | **Error** | **V_P_/V_S_** | **Error** | **Derivation** |
| --- | --- | --- | --- | --- | --- | --- | --- | --- | --- |
| D10 | 34.37 | 97.92 | 4500 | -461.28 | 59.40 | 2.64 | 1.83 | 0.05 | This study |
| MJ22 | 34.89 | 98.21 | 4231 | -397.74 | 60.08 |  | 1.81 |  | Interpolated |
| MJ21 | 35.11 | 98.86 | 4242 | -348.61 | 60.61 |  | 1.79 |  | Interpolated |
| QWA | 35.75 | 98.13 | 3431.7 | -318.27 | 60.93 |  | 1.78 |  | Interpolated |
| ZYU | 35.58 | 98.55 | 3901 | -316.43 | 60.95 | 3.50 | 1.78 | 0.07 | This study |
| QSA | 35.66 | 98.41 | 3707 | -314.74 | 60.83 |  | 1.78 |  | Interpolated |
| XGA | 35.94 | 97.93 | 3174 | -308.58 | 60.39 |  | 1.77 |  | Interpolated |
| DL02 | 35.94 | 97.93 | 3163 | -308.55 | 60.38 |  | 1.77 |  | Interpolated |
| HLM | 35.87 | 98.09 | 3319 | -308.39 | 60.37 |  | 1.77 |  | Interpolated |
| XBA | 36.07 | 97.79 | 2988 | -302.44 | 59.94 |  | 1.77 |  | Interpolated |
| WENQ | 35.42 | 99.44 | 4018 | -292.91 | 59.26 |  | 1.76 |  | Interpolated |
| DL03 | 36.21 | 98.19 | 3330 | -271.25 | 57.70 |  | 1.73 |  | Interpolated |
| XIH | 35.51 | 99.81 | 3808 | -268.46 | 57.50 | 1.50 | 1.73 | 0.03 | Zheng et al., 2016 |
| DL00 | 36.28 | 98.12 | 3225 | -267.28 | 57.41 |  | 1.73 |  | Interpolated |
| DLN | 36.37 | 98.02 | 3072.3 | -263.36 | 57.11 |  | 1.74 |  | Interpolated |
| NW22 | 36.38 | 98.08 | 3097 | -259.28 | 56.80 |  | 1.74 |  | Interpolated |
| DL01 | 36.42 | 98.14 | 3131 | -253.35 | 56.34 |  | 1.75 |  | Interpolated |
| XHAI | 35.60 | 99.99 | 3293 | -251.43 | 56.19 |  | 1.75 |  | Interpolated |
| CHA1 | 36.67 | 98.13 | 3115 | -228.65 | 54.45 | 1.46 | 1.77 | 0.03 | This study |
| CHA0 | 36.63 | 98.24 | 3201 | -228.64 | 54.10 | 1.59 | 1.75 | 0.03 | This study |
| MJ20 | 35.89 | 99.99 | 3270 | -223.85 | 50.40 | 2.31 | 1.72 | 0.05 | This study |
| MJ19 | 35.96 | 100.20 | 3133 | -207.50 | 49.70 |  | 1.74 |  | Interpolated |
| ERTA | 36.01 | 100.32 | 3028 | -197.11 | 49.25 | 1.82 | 1.76 | 0.05 | This study |
| WL00 | 36.92 | 98.49 | 2966 | -189.50 | 48.90 |  | 1.76 |  | Interpolated |
| MJ18 | 36.11 | 100.44 | 2983 | -181.76 | 48.54 |  | 1.77 |  | Interpolated |
| WL01 | 37.05 | 98.38 | 3167 | -180.98 | 48.51 |  | 1.77 |  | Interpolated |
| WL03 | 36.99 | 98.54 | 3069 | -180.70 | 48.49 |  | 1.77 |  | Interpolated |
| MJ17 | 36.22 | 100.53 | 2914 | -167.80 | 47.90 | 2.21 | 1.77 | 0.05 | This study |
| WL02 | 37.29 | 98.39 | 3429 | -157.94 | 47.51 |  | 1.76 |  | Interpolated |
| NW21 | 37.31 | 98.34 | 3477 | -157.73 | 47.50 |  | 1.76 |  | Interpolated |
| MJ16 | 36.35 | 100.66 | 3138 | -148.85 | 47.15 | 2.15 | 1.75 | 0.05 | This study |
| NW20 | 37.37 | 98.84 | 3480 | -131.04 | 62.05 | 2.00 | 1.68 | 0.04 | this study |
| GHTN | 36.39 | 100.83 | 3245 | -137.68 | 49.70 | 2.42 | 1.73 | 0.04 | This study |
| MJ15 | 36.43 | 101.00 | 3333 | -126.76 | 50.64 |  | 1.73 |  | Interpolated |
| MJ14 | 36.52 | 101.12 | 3156 | -112.69 | 51.86 |  | 1.74 |  | Interpolated |
| YK00 | 37.68 | 98.63 | 3646 | -109.71 | 52.12 |  | 1.74 |  | Interpolated |
| MJ13 | 36.62 | 101.22 | 2857 | -98.30 | 53.10 | 3.12 | 1.74 | 0.07 | This study |
| HUN | 36.76 | 101.22 | 2893.5 | -84.82 | 53.32 |  | 1.74 |  | Interpolated |
| LM00 | 37.88 | 98.82 | 3817 | -82.01 | 53.36 |  | 1.74 |  | Interpolated |
| MJ12 | 36.86 | 101.37 | 2694 | -68.50 | 53.58 |  | 1.74 |  | Interpolated |
| MJ11 | 37.00 | 101.45 | 2847 | -52.06 | 53.85 | 1.61 | 1.74 | 0.04 | This study |
| H11 | 38.16 | 99.17 | 4085 | -39.92 | 55.46 |  | 1.68 |  | Interpolated |
| MJ10 | 37.12 | 101.51 | 2753 | -37.02 | 55.85 | 2.04 | 1.66 | 0.05 | This study |
| MJ09 | 37.24 | 101.47 | 2941 | -27.44 | 54.15 | 1.88 | 1.72 | 0.04 | This study |
| QL02 | 38.35 | 99.37 | 3482 | -12.82 | 55.94 |  | 1.70 |  | Interpolated |
| MJ08 | 37.38 | 101.55 | 2897 | -11.14 | 56.15 | 2.00 | 1.70 | 0.04 | This study |
| NW19 | 38.19 | 100.02 | 2917 | -0.11 | 58.09 |  | 1.73 |  | Interpolated |
| MJ07 | 37.50 | 101.57 | 3257 | 1.37 | 58.35 | 2.51 | 1.73 | 0.04 | This study |
| QL01 | 38.46 | 99.54 | 3293 | 4.77 | 57.63 |  | 1.73 |  | Interpolated |
| QL00 | 38.19 | 100.24 | 2735 | 9.12 | 56.71 |  | 1.73 |  | Interpolated |
| NW18 | 38.17 | 100.34 | 2992 | 12.40 | 56.02 |  | 1.73 |  | Interpolated |
| MJ06 | 37.76 | 101.69 | 2937 | 31.93 | 51.89 |  | 1.74 |  | Interpolated |
| SN00 | 38.83 | 99.62 | 2363 | 44.23 | 49.28 |  | 1.74 |  | Interpolated |
| MJ05 | 37.86 | 101.79 | 2672 | 45.58 | 49.00 | 1.99 | 1.74 | 0.04 | This study |
| NW10 | 38.41 | 100.68 | 2551 | 50.00 | 46.84 |  | 1.76 |  | Interpolated |
| SN01 | 38.86 | 99.72 | 2186 | 51.78 | 45.98 |  | 1.77 |  | Interpolated |
| MJ04 | 37.91 | 101.81 | 2566 | 51.84 | 45.95 | 1.98 | 1.77 | 0.05 | This study |
| NW09 | 38.48 | 100.71 | 2274 | 58.26 | 46.34 |  | 1.75 |  | Interpolated |
| NW08 | 38.52 | 100.74 | 2122 | 63.51 | 46.65 |  | 1.74 |  | Interpolated |
| SHAG | 38.01 | 101.95 | 2336 | 67.61 | 46.90 | 2.22 | 1.73 | 0.05 | This study |
| NW07 | 38.56 | 100.78 | 2027 | 68.82 | 51.20 | 2.89 | 1.72 | 0.06 | This study |
| BY00 | 38.96 | 99.94 | 1851 | 70.13 | 50.53 |  | 1.72 |  | Interpolated |
| NW06 | 38.62 | 100.87 | 1933 | 79.05 | 45.93 |  | 1.75 |  | Interpolated |
| NW05 | 38.65 | 100.91 | 1900 | 83.12 | 43.84 |  | 1.77 |  | Interpolated |
| MJ03 | 38.16 | 102.00 | 2108 | 83.68 | 43.55 | 1.88 | 1.77 | 0.05 | This study |
| NJY0 | 39.03 | 100.13 | 1605 | 85.24 | 44.63 |  | 1.76 |  | Interpolated |
| NW04 | 38.71 | 100.99 | 1840 | 92.62 | 49.75 | 2.08 | 1.72 | 0.05 | This study |
| LJP0 | 39.15 | 100.13 | 1449 | 97.13 | 43.75 |  | 1.79 |  | Interpolated |
| JCDC | 38.29 | 102.03 | 1895 | 97.39 | 43.40 | 2.06 | 1.79 | 0.05 | This study |
| NW03 | 38.78 | 101.08 | 1754 | 103.26 | 46.82 |  | 1.75 |  | Interpolated |
| NW02 | 38.82 | 101.05 | 1759 | 106.08 | 48.46 |  | 1.73 |  | Interpolated |
| MJ02 | 38.35 | 102.13 | 1713 | 108.37 | 49.80 | 2.01 | 1.71 | 0.04 | This study |
| NW01 | 38.90 | 101.11 | 2039 | 116.02 | 48.85 | 2.04 | 1.76 | 0.05 | This study |
| XWAN | 38.45 | 102.24 | 1560 | 122.63 | 50.10 | 1.50 | 1.74 | 0.03 | Zheng et al., 2016 |
| NW11 | 38.96 | 101.18 | 1871 | 124.96 | 49.43 |  | 1.75 |  | Interpolated |
| NW12 | 39.00 | 101.20 | 1776 | 129.90 | 48.00 | 1.78 | 1.76 | 0.04 | This study |
| MJ01 | 38.57 | 102.23 | 1472 | 133.20 | 49.75 | 2.34 | 1.66 | 0.05 | This study |
| NW13 | 39.08 | 101.37 | 1545 | 144.64 | 48.51 |  | 1.76 |  | Interpolated |
| LONY | 38.65 | 102.32 | 1410 | 144.97 | 48.52 |  | 1.76 |  | Interpolated |
| MJ00 | 38.76 | 102.35 | 1352 | 157.11 | 48.95 |  | 1.76 |  | Interpolated |
| NW14 | 39.22 | 101.44 | 1423 | 161.50 | 49.10 | 1.72 | 1.76 | 0.04 | This study |
| NW15 | 39.23 | 101.59 | 1417 | 168.98 | 49.25 | 1.89 | 1.75 | 0.04 | This study |
| NW16 | 39.21 | 101.68 | 1563 | 170.92 | 49.22 |  | 1.75 |  | Interpolated |
| ZHJJ | 38.89 | 102.42 | 1337 | 172.51 | 49.19 |  | 1.75 |  | Interpolated |
| MJA1 | 39.03 | 102.50 | 1490 | 189.80 | 48.90 | 1.88 | 1.74 | 0.05 | This study |
| NW17 | 39.34 | 102.09 | 1600 | 201.32 | 49.90 | 2.29 | 1.78 | 0.05 | This study |
| MJA2 | 39.30 | 102.70 | 1246 | 223.63 | 49.35 | 1.73 | 1.80 | 0.05 | This study |

Note: Data marked yellow are interpolated according to their station distances from the North Qilian Suture (NQS), because the Vp/Vs ratios at these stations were not able to be obtained by the h-k stacking, which can be mainly attributed the drastic fluctuation of the Moho topography near these stations. Data marked Zheng et al. 2016 are from (23).
